# Like a hotel, but boring: users’ experience with short-time community-based residential aftercare

**DOI:** 10.1186/s12913-017-2777-z

**Published:** 2017-12-16

**Authors:** Eirik Roos, Ottar Bjerkeset, Margrét Hrönn Svavarsdóttir, Aslak Steinsbekk

**Affiliations:** 1 Department of Public Health and Nursing, Norwegian University of Science and Technology, NTNU, 7491 Trondheim, Norway; 2 Municipality of Trondheim, Norway; 3 Faculty of Nursing and Health Sciences, Nord University, Levanger, Norway; 4 Department of Health Sciences, Norwegian University of Sciences and Technology, Gjøvik, Norway; 5 School of Health Sciences, University of Akureyri, Akureyri, Iceland

**Keywords:** Community residential aftercare, Discharge-ready mental health patients, Severe mental illness, Qualitative study

## Abstract

**Background:**

The discharge process from hospital to home for patients with severe mental illness (SMI) is often complex, and most are in need of tailored and coordinated community services at home. One solution is to discharge patients to inpatient short-stay community residential aftercare (CRA). The aim of this study was to explore how patients with SMI experience a stay in CRA established in a City in Central Norway.

**Methods:**

A descriptive qualitative study with individual interviews and a group interview with 13 persons. The CRA aims to improve the discharge process from hospital to independent supported living by facilitating the establishment of health and social services and preparing the patients. The philosophy is to help patients use community resources by e.g. not offering any organized in-house activities. The main question in the interviews was “How have you experienced the stay at the CRA?” The interviews were analyzed with a thematic approach using systematic text condensation.

**Results:**

The participants experienced the stay at the CRA “Like a hotel” but also boring, due to the lack of organized in-house activities. The patients generally said they were not informed about the philosophy of the CRA before the stay. The participants had to come up with activities outside the CRA and said they got active help from the staff to do so; some experienced this as positive, whereas others wanted more organized in-house activities like they were used to from mental health hospital stays. Participants described the staff in the CRA to be helpful and forthcoming, but they did not notice the staff being active in organizing the aftercare.

**Conclusions:**

The stay at the CRA was experienced as different from other services, with more freedom and focus on self-care, and lack of in-house activities. This led to increased self-activity among the patients, but some wanted more in-house activities. To prepare the patients better for the stay at the CRA, more information about the philosophy is needed in the pre-admission process.

**Electronic supplementary material:**

The online version of this article (doi:10.1186/s12913-017-2777-z) contains supplementary material, which is available to authorized users.

## Background

Collaboration between psychiatric hospitals and primary care services is essential to reduce length of stay and improve follow-up for hospitalized patients [1]. Recent study have found that 86% for individuals with severe mental illness (SMI) were reinstitutionalized over a 7-years follow-up period and 73% were readmitted in the first year after discharge [2]. This indicates that patients with SMI are in need of aftercare [3]. They are also susceptible to ineffective coordination between systems [4, 5]. Therefore, a number of collaborative models between hospitals and primary care have been described in recent years [6].

A review of 21 randomized trials found that a structured discharge plan tailored to the individual patient probably brings about a small reduction in hospital length of stay [7]. Another review found a reduction in readmission between 14% and 37% due to pre- and post-discharge patient psychoeducation, structured needs assessments, and inpatient/outpatient provider communication [8]. There are also a number of interventions used in primary care such as brief motivational interviews [9], peer support and a good therapeutic relationship [10, 11], self-referral inpatient treatment [12], Crisis homes [13] and care management [14–17].

A study from Germany found that the most protective factors for rehospitalization were employment, partnership, and a sheltered living situation [18], whereas the risk factors for increased rehospitalization were urban living and concurrent substance use disorder. Patients with SMI have reported that they see social malaise as an explanation for being readmitted [19], and a qualitative study from the US suggested a history of frequent psychiatric hospitalizations, non-adherence to aftercare treatment, and substance misuse as possible risk factors for readmission [20]. Further, a review of qualitative studies on users’ experience of progress and recovery from critical psychiatric illness during the first month after discharge suggests that patients and their families have a desire for more autonomous control over their own recovery [21].

A collaborative process between patients with SMI, hospitals, and primary care is thus needed to ensure that the most important services are in place immediately after discharge [4]. The patients’ needs for aftercare services provided by primary care can be assessed by the hospital staff prior to discharge [7, 8, 22]. However, personnel in hospitals and community services will likely have different views on what types of services a person with SMI needs in the community. Personnel in the community mental health services often have long clinical experience following patients outside hospital settings, and are likely to be well suited to identify the type of community service needed, even though they do not have specialist-level competency.

One solution to these challenges is to discharge patients with SMI, who need community services, to inpatient short-stay in the community in a step-down model with focus on preparing for independent support living. There are examples of residential community units targeting the discharge process [23, 24]. These mirrors some of the features suggested for alternative hospital care with small units, normalizing facilities, more flexibility and open door and partnering with the community [25]. However, we have not found any studies on community residential aftercare (CRA) units that do not offer organized in-house activities. Such a CRA unit was established in the city of Trondheim in central Norway in 2009. The aim of this CRA unit is to improve the discharge process from hospital to independent supported living by facilitating the process of establishing community health and social services and supporting self-care, community engagement and not offering organized in-house activities to ensure community orientation among the patients.

Therefore, the aim of this study was to explore how patients with SMI in need of community support after a mental health hospital stay experienced the stay in the CRA unit established in the City of Trondheim in Central Norway.

## Methods

This was a descriptive qualitative study using nine semi-structured face-to-face individual interviews followed by one group interview to validate and expand on the findings from the individual interviews. Qualitative methods are well suited for research relating to individual experiences and perceptions [26]. The individual interviews were conducted between May 2013 and May 2015, and the group interview was held in June 2016.

### Ethical considerations

The study was conducted in accordance with the Declaration of Helsinki and was approved by the regional Committee for Medical Research Ethics in Central Norway (2011/1770). The participants received written and oral information about the study, and they were informed that they could withdraw at any time. Written consent was obtained before the interviews were conducted, and confidentiality was assured.

### The CRA service

The study took place in a community residential aftercare (CRA) service in Trondheim, a city in Central Norway with 190,000 inhabitants. In September 2009, the municipality established the short-stay CRA for patients with SMI discharged from the mental health department at the local university hospital. One aim of the CRA was to reduce the time the patients, who normally would be in need of public community services after discharge, spent in the mental health hospital after they are been declared ready for discharge. This is done by supporting self-care and facilitating community health and social services, as described below.

For the patients to be eligible for discharge to the CRA, the hospital must have assessed and document that the patients are ready for discharge, such as deciding on the main diagnosis and starting a treatment plan. The patients are usually transferred on the same or following day after the hospital have contacted the CRA. The stay at the CRA is voluntary, meaning that the patients can leave any time they want to. The tentative length of stay in the CRA is up to 4 weeks, based on experiences with the time it takes to organize public community services that the patients’ needs once, when living at home. The length of stay is usually longer for homeless patients’ due to the practicalities of making housing arrangement. In 2016, the average of length of stay at the CRA was 37 days (69 patients), 64 days for homeless (14 patients) and 29 days for those with a residence (55 patients).

The CRA has 14 single rooms with their own TV and bathroom, 10 reserved for discharge-ready hospitalized patients (step-down), and for patients living in the community who are homeless or need another residence (step-up), two rooms reserved for self-referral patients who have previously been at the CRA, and two rooms reserved for sub-acute admission directly from patients’ residence (step-up). There are also three single rooms not in use. Patients using beds as part of step-up were not part of this study. There are common rooms and kitchen where the patients can make their own food, whenever they want.

The CRA operates 24/7 and is staffed with psychiatric nurses, general nurses and nursing assistants. All except one have experiences from community services to ensure their understanding of the need a patient can have in the community. Four employees are present during the day, two at the evening shift and one nurse during night shift. In addition, a team leader is present at daytime on weekdays. The nurse on the night shift can alarm for assistance from nearby services. The staff has training in recovery-oriented strategies, such as self-management and self-responsibility to manage daily activities. A general practitioner (GP) is present in the CRA 1 day a week and offers a consultation to all patients who have recently been admitted, and those in need of medical follow-up at the CRA. The GP cooperates with the patients’ regular GP and requests the patients to make regular appointment with these.

To prepare patients for independent supported living the patients are directed to activities in the community. The philosophy of the CRA is to purposively not offering any in-house activities. Instead, the patients are informed about activities in their neighborhood and in the community. Thus, there are no organized activities at the CRA like common meals, therapy options or equipment for exercise. Consequently, there is a strong emphasis on and practical training to support self-care; how to structure daily routines including sleep patterns, strategies to cope with difficult symptoms, personal hygiene, appointments with other agencies, self-care and independent living like use of public transport, shopping, meal planning and social and leisure activities outside the CRA. The patients also have overnight stays in their own home during the stay at the CRA.

The CRA also is central in facilitating the process of establishing community health and social services to support the transition from the hospital to independent supported living. When patients arrive, they get a dedicated contact person whose main responsibility is to support the patient during the whole stay. The contact person also observes and assesses the patient following a checklist presented in Table 1, always with a focus on preparation for the discharge process from the CRA. During the stay, the result of the individual assessment is discussed with the patient and communicated to the community Health and Welfare agency services to help them agreeing on the level of services to be provided after discharge, e.g. housing for homeless, relocation (move away from a substance abuse neighborhood), home nursing services and home care services. This is done in meetings, coordinated by the CRA, between the patient and the agencies that offer the different types of services that are judged to be appropriate. The process is started as early as possible to establish relationship between the patient and the service providers offering the follow-up services after discharge.

**Table 1:** Checklist for observation and assessment of patients used by the staff during the CRA stay in preparation for the discharge planning process and to help decide on the type of services to be offered afterwards

Area	Cues
Self-care	Hygiene, food preparation, diet, cleaning, washing, shopping, exercise/activities, and mastering substance abuse problems
Medicating	Self-medicating, misuse of medicines, need of support with medicating
Economy	Assess needs of any support to manage finances, e.g. pay bills
Social network	Assess the social network, relationships, and participation in any social activities
Housing	Visit the residence together with the patients – assessment of the facilities in the residence, such as cooking and cleaning
Primary care services	Assess present follow-up services and other tailored services
Leisure time	Assess patients’ hobbies and interests
Facility	Assess patients’ technical aid needs
Mobility	Assess the need for assistance to take the bus, visit public offices, cultural and leisure activities
Job/education	Assess present education and job – arrange job/education or activities together with the patients
Before discharge	The contact person organizes a meeting with the patient and community agencies for assessment and approval of tailored services. The follow-up services must be up and running at home at the expected date of discharge. The general practitioner has received discharge summary from the CRA.

Before discharge from the CRA, patients receive information about the possibility of later self-referral to a short (maximum of 3 days) inpatient stay at the CRA.

### Sample and recruitment

The aim was to recruit patients with SMI currently staying at the CRA or who had been discharged from the CRA within the last 4 months. Participants were selected to ensure variation in age, gender, and time from admission to the CRA or time since discharge from the CRA.

To recruit participants for the individual interviews, the team leader in the CRA introduced the study to eligible participants at the CRA both orally and by handing out invitation letters. Eligible participants who had been discharge were contacted by phone. The CRA staff passed on contact information for those who wanted to participate to the first author (ER). Then, the first author contacted the participants by phone and repeated and gave more information about the study. Patients were given the choice to be interviewed in their own apartment, in a public office, or in the CRA.

To recruit patients for the group interview, the team leader in the CRA handed out invitation letters to eligible participants at the CRA and scheduled the interview. The group interview was conducted in a common room at the CRA.

### Data collection

The individual interviews were conducted by the first author, and the group interview by the first (ER) and the fourth author (AS). The staff in the CRA did not take part in any of the interviews, but a contact person was present at one individual interview at the patient’s request. The interviews were audiotaped and transcribed verbatim. The average time of the individual interviews was 27 min (range approx. 15–45 min), and the group interview lasted 1 h and 47 min.

An interview guide (Additional file 1) was used in all interviews to ensure that all participants were given the opportunity to comment on the same topics. The main question was “Can you tell me/us about your experience with your stay at the CRA?” The follow-up questions addressed what the participants were most and least satisfied with, their daily activities during the stay, and how they perceived the organization of services they would need after discharge. Those who had used the self-referral inpatient care were asked about their experience with this particular service.

### Analysis

The data were analyzed following systematic text condensation, which is a method suited for thematic cross-case analysis inspired by Giorgi’s psychological phenomenology approach [27, 28]. The analysis started after the first four interviews were done and continued simultaneously with the recruitment and interview process. The recruitment continued until no new themes emerged from the analysis and the material was considered saturated.

The analysis itself was also iterative, meaning that the four distinct steps of systematic text condensation were repeated during the process. The first step was to read the transcribed interviews with an open mind to obtain a general impression and to identify preliminary themes. The first author read all interviews and selected, based on richness, two individual interviews that all authors read. In the second step, the transcripts were systematically reviewed line by line to identify meaning units, which were classified and sorted into the preliminary themes. Particularly at this step, the authors had several meetings to discuss and refine the subthemes and themes. In the third step, the meaning units within each subtheme, established in the second step of analysis, were reduced into a condensate, an artificial quotation maintaining, as far as possible, the original terminology applied by the participants. This facilitated further sorting between the subthemes. In the fourth and last step, the condensates of each subtheme were rewritten in general descriptions, and the final sorting of subthemes into the main themes was finalized.

The main part of the analysis was performed by the first author and discussed with the co-authors. The analysis was further validated by a thorough review of the original transcript of each interview to ensure all points of significance were reflected in the results. The quotations that best illustrated the themes were chosen to support the results. The description of the chapter “The CRA service” was validated by the manager and the team leader of the CRA to ensure that the authors had understood the purpose and the philosophy behind the CRA.

## Results

A total of 15 patients with SMI were approached, and of these 13 participants were interviewed (Table 2), nine in individual interviews (seven men and two women) and four participants in a group interview (three men and one woman). Four participants were interviewed in their own apartment, seven in the CRA, and two in a public office.

**Table 2:** Characteristics of the participants (*n* = 13)

Characteristics	*N* (%) or Mean (SD, range)
Gender	
-female	3 (23%)
-male	10 (77%)
Age	
-Mean age in yrs. (SD, range)	42 (13.0, 20 to 63)
Living situation	
-homeless	3 (23%)
-living alone	11 (85%)
-with wife/husband/live-in partner	1 (8%)
-with children	2 (15%)
Employment status	
-full-time employment	1 (8%)
-unemployed	8 (62%)
-disability pension	4 (31%)
Main diagnosis - ICD-10 code	
-mental and behavioral disorders (F10)	2 (15%)
-schizophrenia, schizotypal, delusional disorders (F20)	2 (15%)
-mood (affective) disorders (F30)	5 (38%)
-behavioral and personality disorders (F60)	3 (23%)
-Other organic personality and behavioral disorders due to brain disease, damage, and dysfunction (F07.8)	1 (8%)
Length of stay at the CRA	
-median (range) and mean (SD) duration in weeks	5 (3 to 24), 8.9 (7.4)^a^
Time of interview	
-during the stay	7 (54%)
-after discharge	6 (46%)

^a^The reason for the high mean were three homeless patients staying respectively 19, 21 and 24 weeks waiting for residence. The range in length of stay without these participants was 3 to 8 weeks and the mean 5.2 weeks

The findings were categorized into five themes: 1) Not what I expected; 2) Like a hotel, but boring, 3) Treatment, a place to rest, or preparation for independent living? 4) Coordination with other agencies; and 5) Use of self-referral stay.

### Not what I expected

Whether the participants appeared to have understood the philosophy behind the CRA seemed to influence their attitude and experience. Some clearly expressed that they did not expect anything from the staff and were happy with that, whereas others wanted a different type of service than the one the CRA offered. Some participants said they were not informed about what they could expect at the CRA before they arrived. They said that they had only been informed that they would be discharged to a residential institution in primary care settings and expected something like a mental health hospital. Others said they had received information about the CRA, but mostly about practical aspects, like that they would have a single room and a safe place to stay.

*“I got information from the staff in the mental hospital about the stay at CRA and was told they offer a single room, serve dinner, and there were staff 24/7. Otherwise, there was no detailed information given.”*

None of the participants clearly stated that they had received information that the CRA did not offer treatment or in-house activities, nor were they informed that they were expected to be active in finding their own recreation in the community. Typically, the participants received this information when they arrived at the CRA from the staff who welcomed them and gave them information about the stay. Nevertheless, some participants that had stayed for a while in the CRA still spoke about it in a way indicating that they expected the services they were used to from the mental hospital.

*“I felt the stay was just as a place to rest.”*

### Like a hotel, but boring

All the participants expressed great satisfaction with the freedom and privacy they had at the CRA, especially that they could leave the residential care whenever they wanted without asking for permission, since the staff trusted them.

*“[You] can go in the fridge and prepare food for yourself whenever you want. It's just to let the staff know when you go for a walk — no begging.”*

All the participants agreed that their stay at the CRA was different from other mental health institutions they had experienced. One participant compared the stay at the CRA with a hotel stay. This was supported by others, such as with descriptions of freedom and having their own private room, where they could do what they wanted without interruption. Several spoke about the advantage of having a private TV in their room, which gave them the possibility of choosing their own TV programs. This was compared with institutions that had only one TV in a common room, with frequent discussions regarding which program to watch. Other characteristics of the CRA being like a hotel were the quiet and relaxed atmosphere without disturbance from other patients and deciding when to be alone, relax, and do activities.

*“You feel that you are in a nice hotel, but nothing else.”*

However, as the previous quote illustrates, some participants experienced the stay as boring. It was clear from the interviews that the freedom and lack of in-house activities came at a price for several of the participants. They talked about their previous experience where they were encouraged to interact with other patients and to participate in organized in-house activities like common meals, group therapy, walking tours, bowling, or the cinema. Several participants said that they missed these types of organized group activities, and some said that they did not understand why this was not offered at least to some extent at the CRA. Even when asked in detail in the interview about their understanding of the CRA not offering in-house activities in order to encourage them to be active themselves, some maintained that there should be at least some activities. They argued that they either did not feel they had other activities in the community to take part in or that there were some days they did not feel like going out due to how they felt.

*“I thought there would be more in-house activities, but there were none. So, you felt in a way that you were just sitting there waiting with nothing to do or that someone would try to take you out or get you to participate in normal life. You were on your own. The staff asked me how I was going to spend the day and I had to find out what to do.”*

### Treatment, a place to rest, or preparation for independent living?

Participants’ expectations concerning treatment at the CRA varied. Some said they were used to conversational therapy from their previous inpatient stay and that they felt a continuing need for this in the CRA. One participant said that despite recommendations from the hospital, he was not offered conversational therapy as part of the stay at the CRA. This participant expressed that the staff had failed in taking the responsibility to help him get appropriate treatment at both the CRA and outside.

*“I was not offered conversational therapy, and there was no treatment.”*

In contrast, some participants said that the staff observed them without being intrusive, and they were confident that the staff would act if someone needed further treatment.

*“It was only when I came to the residential care and got relaxed that I realized how tired I was. To have the opportunity only to withdraw, with no requirement to participate in activities, made me see my situation more clearly.”*

All participants reported some positive experiences with the staff at the CRA. They especially appreciated getting a designated contact person. They compared this to their prior experiences from hospitals, where they sometimes had various “contact persons,” who all had the same level of responsibility. This was reported to result in a lack of coordination between the different contact persons, misunderstandings, and sometimes inadequate help. Some stated that the staff in the CRA was more “hands-on” and solution-oriented in helping with practical and concrete tasks than the hospital staff. Some participants said that the contact person really wanted to help them, as they felt that the staff took personal responsibility for them during the stay.

There was variation to what degree the participants said that the staff activated them. Most said that they received information from the CRA staff about various activities in their own neighborhood, such as fitness centers, low-threshold services, and cultural and leisure activities, or were encouraged to take up their previous activities.

*“They also teach me how to structure my life by finding housing and facilitate daily activities like cleaning and preparing meals.”*

However, some felt that the staff went too far in wanting the participants to become active themselves. One participant told about how he had asked the staff to accompany him to an activity center but they only encouraged him to go by himself.

*“An employee from the mental health services [ambulant team, not part of the CRA] followed me to the activity center the first and second time I was there. Later, because of that support, I could go there by myself. So, it was very important for me that someone escorted me the first time and showed me the place so I felt safe.”*

### Coordination with other agencies

Most participants said they had been in meetings with community services where they discussed what type of services they needed after discharge from the CRA. Some participants said that the contact person had initiated such meetings, but in general the informants did not talk about the CRA as having an active role in planning their aftercare.

When asked about the meetings they had with the community services, the participants mostly remembered being asked which practical services they needed in order to manage everyday life at home. Some described their need for continuing conversational therapy and complained that there was no information on how to get help to cope with their mental health problems. Other participants said that they talked to CRA staff about preparing themselves before meetings with other community agencies.

*“Yes, an employee from the Health and Welfare office came and talked to me, and then I was offered a follow-up service that I still use, as well as help from the Child and Family agencies.”*

Some of the participants did not know whether the services they received prior to hospitalization were still available to them after their inpatient stay. It was said that they missed this contact and experienced a kind of discontinuity between the staff in the primary care and the specialist from the hospital. They said it would have been an advantage if the primary care staff they were used to at home also could visit them during the stay at the CRA.

*“I have a good relationship with an employee in a community ambulant team, and I would have liked to keep contact with him while I was hospitalized. I would like to have a meeting with him once a week, as well as cooperation with the staff in the mental hospital.”*

The six participants who were interviewed after discharge from the CRA talked about similar experiences regarding preparation for their home situation. They all expressed satisfaction with the services they were offered after CRA discharge and said that meetings with different agencies during the stay in CRA were essential in setting this up. They told that they had been asked about their own needs, and some had ended up with different types of services than they had before their admission at the mental hospital. Examples of changes were from having had only Community mental health consultation, in addition they were offered services from the Agency of Children and Family Affairs, Day center, Assertive Ambulant Team (ACT-team) and self-referral at the CRA.

Three of the participants interviewed after discharge reported that they continued with activities they were introduced to during their stay in CRA, such as work experience at a private firm, volunteering in a church organization, and visiting a low-threshold day center. They found that planning the discharge process was different in the CRA compared to their prior experience with planning the discharge process from a mental hospital. In the CRA, they were asked which services they needed in order to manage their everyday life at home, such as follow-up visits from community agencies, and the Norwegian Labor and Welfare Administration (NAV) arranged work practice for them.

*“When I was at the residential care, they arranged a new system for me together with other agencies of the municipality.”*

### Use of self-referral stay

Nearly all patients said they were pleased with the possibility to self-refer from home for a short stay at the CRA. A typical comment from those who had been discharged from the CRA and who had used the self-referral was that it made them feel safe to know that they had the CRA to contact. One participant who had used the self-referral once talked about how the staff had encouraged him to try to stay at home a bit longer when he called and asked to be admitted. This made him aware of the importance of trying to postpone using the service.

*“When I'm home I push myself further and further and wait with requesting self-referral stay, so it will not be so often.”*

Those who had used the service said that they used it to get “back on track” again (e.g. re-establish daily routines like sleep pattern). They experienced the short self-referral stays in the CRA as a good way to stabilize their situation and relax. Some said that without the offer in the CRA they would have had more mental health hospital admissions, which they wanted to avoid.

*“Self-referral stay at the CRA is much better than an ‘emergency’ admission at the hospital. In and out of the emergency department and then home again does not help.”*

Another participant felt that it was good to use self-referral stay for one or 2 days to relax and regain energy but would not want to stay there longer, because there were no organized activities offered.

## Discussion

The main findings of this study were that the participants experienced the stay at the CRA like a hotel, but that it was boring. There were participants who did not agree with the philosophy of the CRA of not offering in-house activities, and they felt that it would have been better to have some activities. All participants said they appreciated the freedom to structure their daily routines without regulations. The participants reported that they had discussions with the CRA staff about how their services should be organized after discharge, but in general they did not acknowledge that the CRA had an active role in facilitating this.

### What to do at the CRA?

We found that not all participants understood or agreed with the approach taken at the CRA of not offering organized in-house activities in order to encourage self-directed activities. The CRA’s philosophy to not offer organized in-house activities is to prevent patients from being inactive and institutionalized [29]. A review concluded that it is essential that patients understand to the importance of self-care and to formulate goals for the stay [30]. Also, a Swedish 2-year follow-up study among 49 persons with SMI reported that self-formulated rehabilitation goals are important in daily activities [15]. However, the participants in this study lacked information about the CRA and didn’t feel prepared for the stay. In the future, this information should be given well ahead of discharge from the mental hospital. This is especially important, as it is likely that participants’ expectations of the offered services were influenced differently, also depending on their diagnosis, ranging from personality disorders to organic brain diseases. Thus, as patients with SMI constitutes a heterogeneous group, the degree of self-directed activities and information to clarify the expectations of the services the CRA offers, should be individualized.

Some participants in our study said they took part in activities outside the CRA after some weeks, as a direct consequence of a boring stay, due to lack of in-house activities at the CRA. This indicates that the philosophy of the CRA can help patients to start activities in the community that they can continue after discharge. This is supported by a Danish study that found that community residential facilities were better able to promote residents’ activities both within the facility and in the community than hospital-based psychiatric rehabilitation units [31].

The participants were satisfied with the freedom they experienced at the CRA. However, too much freedom could be problematic for some, as a previous study on patients with schizophrenia found that they spent a large amount of time engaging in passive activities like watching television or sleeping [32]. Therefore, the staff should be aware of the dilemma of supporting patients to rest or supporting them in a recovery orientation to engage in activities [33].

The CRA offered only single rooms where the patient could withdraw when he or she wanted, and this was mentioned by the participants as an important aspect of freedom. Our results are in line with a Danish study on Crisis houses also offering single room [13] where patients’ experienced that they lived a normal life and were seen as humans – not patients. Previous studies have found that overcrowding in psychiatric wards may be associated with increased risk of anxiety and aggression in patients with SMI [34] and that a single room and fewer patients in common rooms in wards lead to reduced levels of stress, pain, and anxiety [35, 36]. Further, our results are keeping with a narrative review [37] that found that well-designed interior settings play an important role in the healing process of patients in health care facilities. A review on the quality of institutional mental health care concluded that an ideal institution should be small and community-based and maximize flexibility, privacy, engagement, and positive therapeutic relationships [38].

### Coordination of services before discharge

The participants said they appreciated having a dedicated contact person in the CRA. Several studies have demonstrated the importance of continuity in the staff–patient relationship to achieve good results in treatment and follow-up [39–42]. However, this must be balanced against the problem arising if the patient develops a dependency relationship with the contact person [14]. Having a dedicated contact person at the CRA could create such dependency, but findings from this study did not indicate that this contact was a barrier to development of a relationship with community staff. However, as participants talked about not maintaining contact with the service providers they had before their hospitalization, more emphasis should perhaps be placed on facilitating regular contact with community service providers during the stay at the CRA. This could contribute to a stable relationship with the career in the community services [43].

In line with our findings, a qualitative study in a psychiatric emergency department in the US also found that it was beneficial to have a care manager to assist patients with primary care connections [14]. Previous studies have also pointed out the importance of involving and listening to patients’ voices in the admission or discharge process [43, 44]. Thus, the approach of the CRA has support both among the participants and in the literature. Nevertheless, given the focus at the CRA of facilitating aftercare, it is surprising that the participants in our study generally did not acknowledge that the CRA had an active role in this, although some said that the contact person had initiated meetings.

### Strengths and limitations

To the best of our knowledge, this is the first study to explore the experience of a stay at a CRA not providing organized in-house activities for patients with SMI. It is also a strength that the participants interviewed during the stay reflected on the services they were just offered, and the participants interviewed after discharge at home could reflect on how their stay had an impact after discharge. We also achieved good variation among the informants, but there might have been patients at the CRA who did not want to be included in the study but could have had other experiences. Further, the psychiatric diagnosis differed across the sample. Therefore, it could be difficult to recommend if there are patients with specific diagnoses that benefit from a stay in the CRA.

## Conclusion

The stay at the CRA was clearly experienced as different from other services, with more freedom and focus on self-care, and lack of in-house activities. This led to increased self-activity among the patients, yet some wanted more in-house activities at the CRA. To prepare the patients better for the stay at the CRA, more information about the philosophy is needed in the pre-admission process.

## Additional file

Additional file 1: Interview guide. (DOC 31 kb)

## Abbreviations

CRA: Community residential aftercare
SMI: Severe mental illness, typically people with a diagnosis of schizophrenia, schizoaffective disorders, major depression, or personality disorders
