# Supplementary material for: Like a hotel, but boring: users’ experience with short-time community-based residential aftercare
Source: BMC Health Serv Res. 2017 Dec 16;17:832. doi: 10.1186/s12913-017-2777-z (PMC5732432; doi:10.1186/s12913-017-2777-z)
Supplement: Additional file 1: — Interview guide. (DOC 31 kb) [file 12913_2017_2777_MOESM1_ESM.doc]

**INTERVIEW GUIDE**

(Individual interviews and a group interview with inpatient in a short-stay community residential aftercare).

The purpose of this study is to explore how people with severe mental illness (SMI) experience a stay in a community residential aftercare (CRA), which aim at improving the discharge process and facilitate the process of establishing health and social services. In addition, the users’ experiences with the tailoring services to their needs and their experiences with using the short stay self-referral inpatient services provided by the CRA.

**Main team**

**Experiences with the stay at the CRA**

1. Could you please tell me how and from whom you get information about the CRA?
   - Do you get any information during the stay in the hospital and by whom?
2. What kind of information was given?
   - About facilities?
   - The purpose with the stay?
   - The length of the stay?
3. After admission in the CRA
   - What kind of information do you get?
   - Was this information in accordance with the information given from the hospital?
   - Information about how to manage the meals?
   - Information about activities in the CRA and outside the CRA?
   - Information about relationship to the staff and to other patients?
   - Information about the self-referral stay at the CRA?
4. Could you please tell me about how you experience the facility at the CRA?
   - Single room – common room - kitchen
5. Could you please tell about how you experience the atmosphere in the CRA?
   - Noisy – hectic – lot of patients(crowded) – rush versus tranquil - quiet – relaxed atmosphere?
   - Could you leave the CRA during the stay whenever you want? Or do you need permission from the staff?
6. Relationship to the staff at the CRA
   - Do you get more than one contact-person?
   - How was the relationship with the contact-person?
   - Could you tell about your experience in the discharge planning process?
     - Users involving in assessing services in the CRA?
     - Users involving in self-formulating goals for the stay?
7. Relationship to other agencies during the stay at the CRA? (Mapping needs of services after discharge (at home))
   - Could you please tell about how these mapping took place?
   - Who was assessing needs?
   - Was it separate meetings with some agencies? Or other meetings?
8. Could you please tell me about yours experiences with the stay at the CRA?
   - Do you experience the stay at the CRA as you were informed?
   - Can you please describe what to do in a day / a week?
9. Could you please tell about the most important you get support within the CRA?
   - Stabilizing treatment – better mental health - no change.
   - Getting other services at home after discharge from the CRA?
10. Could you please tell about why it is important?
    - Involved in the discharge planning process?
    - Prepared for the discharge?
11. Could you please tell if there was anything you missed in the CRA?
    - Organised activities in the CRA? Which?
    - Could you tell why these activities would have been important?
12. Could you please tell about the main differences between a stay in a mental hospital versus a community residential aftercare?

**Open question**

Could you please tell if there are other themes, other than what we have talked about?

**In addition, for participants interviewed after discharge:**

1. Could you please tell about if the stay at the CRA was importance regarding to manage everyday life the first month at home?
   - Experience with the services?
   - Managing daily activities?
   - Experience of safety?
   - When you notice symptom change/increase – how do get access to help?
   - Experience with the contact-person in the follow-up care?
2. Experience with use of self-referral stay at the CRA?
   - Why do you used self-referral stay?
3. What are you most satisfied with regarding to:
   - Housing
   - Job – leisure activities – social network – services from primary care – services from the specialist.
4. What are you not satisfied with regarding to:
   - Housing
   - Job – leisure activities – social network – services from primary care – services from the specialist.

**Open question**

Could you please tell if there are other themes, other than what we have talked about?
